# Gossypol has anti-cancer effects by dual-targeting MDM2 and VEGF in human breast cancer

**DOI:** 10.1186/s13058-017-0818-5

**Published:** 2017-03-09

**Authors:** Jing Xiong, Jiansha Li, Qin Yang, Jun Wang, Tiefen Su, Sheng Zhou

**Affiliations:** 1 Institute of Pathology, Tongji Hospital, Tongji Medical College, Huazhong University of Science and Technology, Wuhan, 430030 China; 2 Department of Pathology, School of Basic Medical Science, Huazhong University of Science and Technology, Wuhan, 430030 China

**Keywords:** Breast neoplasms, MDM2, VEGF, Gossypol

## Abstract

**Background:**

Mouse double minute 2 (MDM2) and vascular endothelial growth factor (VEGF) are important molecules involved in tumor progression. We researched potential inhibitors that simultaneously target MDM2 and VEGF. In our recent study involving the performance of high-throughput screening with a fluorescence polarization assay, gossypol was identified as one of the top hits that inhibit protein-RNA binding activity. Because MDM2 is an RNA-binding protein and its targets include VEGF mRNA, we investigated whether gossypol has an inhibitory effect on MDM2-VEGF.

**Methods:**

UV cross-linking and RNA binding assay, isothermal titration calorimetry assay, and ubiquitination assay were performed to determine mechanisms by which gossypol functions as a dual inhibitor of MDM2 and VEGF. The effect of gossypol on MDM2 and VEGF expression, cancer cell apoptosis, tumor growth and VEGF-mediated angiogenesis were studied in vitro and in vivo in different human breast cancer models with a different p53 status.

**Results:**

We observed that gossypol inhibited expression of both MDM2 and VEGF in human breast cancer cells with either wild-type or mutant p53. A nechanistic study further demonstrated that, through disrupting the interaction between MDM2 protein and VEGF mRNA, gossypol induced MDM2 self-ubiquitination and decreased VEGF translation simultaneously, which resulted in both apoptosis and anti-angiogenesis effects. In vitro, regardless of p53 status, gossypol induced cancer cell apoptosis. In nude mouse xenograft in vivo models, gossypol suppressed tumor growth and VEGF-mediated angiogenesis.

**Conclusion:**

Gossypol has anti-cancer effects by dual-targeting MDM2 and VEGF in human breast cancer. Our study reveals a novel mechanism by which gossypol functions as an anticancer agent. We believe that MDM2-VEGF targeting represents a novel strategy for improving cancer outcome.

## Background

Mouse double minute 2 (MDM2) and vascular endothelial growth factor (VEGF) are important molecules involved in tumor progression. Increasingly there has been a broad consensus that they should be used as key targets for drug development. For example, selective small-molecule MDM2 antagonist nutlin-3a has been discovered and is under pre-clinical development [1]. The recombinant humanized VEGF monoclonal antibody, bevacizumab, is recommended by the National Comprehensive Cancer Network (NCCN) guidelines for treatment of metastatic cancer [2].

MDM2 is a multifunctional oncoprotein that is overexpressed in a variety of human malignancies, including breast cancer [3–9]. Recently published studies, including ours, demonstrate that MDM2 is able to interact with specific RNA sequences or structures [10, 11]. The COOH-terminal RING finger domain of MDM2 protein was found to bind to the AU-rich sequence within the 3′untranslated region (3′UTR) of VEGF mRNA, to stabilize VEGF mRNA, and to increase its translation [12].

MDM2 and VEGF have been considered as potential cancer targets. Various MDM2 inhibitors have been found to have anticancer activity. The most promising of these is nutlin-3a. However, the majority of these inhibitors exert their effects through restoring the p53 apoptotic response [13]. But actually, more than 50% of all solid tumors carry the p53 mutation [14], and there is a higher incidence of p53 mutation or loss of p53 observed in aggressive cancer [15]. Moreover, activated p53 in turn induces MDM2 expression through the p53-MDM2 feedback loop. These compounds do not decrease expression of MDM2 and may even increase it. In light of the p53-independent mechanism through which MDM2 regulates VEGF-mediated tumor angiogenesis, no significant VEGF inhibition or anti-angiogenesis activity has been observed after treatment with nutlin-3a [16].

Bevacizumab is the first Food and Drug Agency (FDA)-approved anti-angiogenic drug for clinical use. There is preliminary evidence of its efficacy when combined with cytotoxic agents. However, even low-dose bevacizumab is associated with increased risk of venous thromboembolism and some patients with a clinical response to VEGF blockade ultimately develop progressive disease [2]. Therefore, numerous studies remain committed to testing novel anti-cancer drugs targeting MDM2 and VEGF, or both simultaneously.

Gossypol, a polyphenol derived from cotton seeds, exhibits potent anti-cancer activities and has completed phase II clinical trials for treatment of human prostate cancer, with promising initial results [17]. However, information on its mechanism is still limited [18–21]. In our recent study involving the performance of a high-throughput screening (HTS) with fluorescence polarization (FP) assay in four chemical libraries containing a total 141,394 known reagents, drugs, and small-molecule compounds, gossypol was identified as one of the top hits that inhibit protein-RNA binding activity [22]. Because MDM2 is an RNA-binding protein and its targets include VEGF mRNA, we investigated whether gossypol has a similar inhibitory effect on MDM2-VEGF. We found that gossypol is a potent inhibitor of MDM2-VEGF. Through disrupting the interaction between MDM2 protein and VEGF mRNA, gossypol induces MDM2 self-ubiquitination and decreases VEGF translation simultaneously, which results in not only cancer cell apoptosis but also suppression of tumor angiogenesis. Our study helps to elucidate the mechanism by which gossypol functions as an anticancer agent. We believe that MDM2-VEGF targeting represents a novel strategy for improving cancer outcome.

## Methods

### Cell lines and reagent

Five human breast cancer cell lines (MCF-7, ZR-75-1, MDA-MB-231, MDA-MB-468 and T47D) were used in this study. The first two have wild-type (wt) p53, while the remaining three are p53-mutant. All five cell lines were obtained from the China Center for Type Culture Collection (CCTCC). The gossypol was purchased from Sigma-Aldrich (St. Louis, MO, USA), and stock solution was prepared at 10 mM in dimethyl sulfoxide (DMSO). In vitro biological activity of gossypol was determined after incubation at various concentrations (2.5, 5.0, 7.5, and 10.0 μM) for different periods of time (2, 4, 8, and 24 h). Equivalent concentrations of DMSO were used as vehicle controls.

### Plasmids and gene transfection

MDA-MB-468 cells with MDM2 knockdown or overexpression were described previously [23]. To determine whether gossypol-induced ubiquitination of MDM2 required the intrinsic self-ubiquitination E3 ligase activity of MDM2 itself, a QuickChange Site-Directed Mutagenesis Kit (Stratagene, La Jolla, CA, USA) was used to mutate MDM2 464; this leads to cysteine being substituted by alanine to generate the plasmid pCMV-MDM2 C464A, which exhibits loss of E3 ligase activity. MDM2 gene promoters 1 and 2 were cloned into the pGL3-Basic vector to generate the MDM2 p1 and p2-Luciferase plasmids. MDA-MB-468 cells were transiently transfected with these MDM2 promoter luciferase plasmids, treated with gossypol and then prepared for testing the luciferase activity of MDM2 promoter reporters using the Dual-Luciferase Reporter Assay System (Promega, Madison, WI, USA) according to the manufacturer’s instructions.

### Quantitative RT-PCR

Quantitative RT-PCR was used to measure the expressions of MDM2 and VEGF at mRNA level. The primer sequences were as follows: MDM2 forward 5′-TGTTGGTGCACAAAAAGACACTT-3′, MDM2 reverse 5′-GCACGCCAAACAAATCTCCTA-3′; VEGF forward 5′-GATGAAAGGCGGCATACGG-3′, VEGF reverse 5′-CAGGGCTATTCTTCTTAGTGTGC-3′.

### Western blot

The protein expression levels of MDM2, p53, VEGF, caspase-3, cleaved caspase-3, and poly(ADP-ribose) polymerase (PARP) were analyzed by western blot. The following antibodies were used: anti-MDM2 antibody (1:1000; Sigma), anti-p53 antibody (1:1000; Santa Cruz Biotechnology, Santa Cruz, CA, USA), anti-VEGF antibody (1:200; Santa Cruz Biotechnology), anti-caspase-3 antibody (1:1000; Santa Cruz Biotechnology), anti-cleaved caspase-3 p11 antibody (1:1000; Santa Cruz Biotechnology), and anti-PARP antibody (1:1000; Cell Signaling, Danvers, MA, USA).

### In vivo protein-RNA binding assay

To detect in vivo binding between MDM2 protein and VEGF mRNA, VEGF mRNA was co-immunoprecipitated with MDM2 antibody from whole-cell extracts by using a previously described method [12]. The resulting RNA was analyzed by quantitative RT-PCR using VEGF-specific primers.

### UV crosslinking and RNA binding assay

The DNA template for synthesis of the VEGF 3′UTR probe was generated using the following sequence: 5′-TAATACGAGTCACTATAGGGAAATTCTACATACTAAATCTCTCTCCTTTTTTAATTTTAATATTTG-3′. This DNA fragment incorporated the T7 promoter sequence (underlined). Internally labeled RNA probes were synthesized by in vitro transcription with T7 polymerase (MAXIScript T7 RNA polymerase kit, Ambion) in the presence of [α-^32^P] UTP (Amersham). GST-MDM2 protein was mixed with ^32^P-labeled probes in the presence or absence of gossypol. Then UV crosslinking of the RNA-protein complexes formed was done using a 254-nm UV light source set at 400,000 μJ/cm^2^. Finally, the UV-irradiated RNA-protein complexes were treated with RNase T1, resolved by 10% SDS-PAGE gel and visualized by autoradiography.

### Isothermal titration calorimetry (ITC) assay

The binding activity of gossypol to either MDM2 RING protein or VEGF 3′UTR was examined by ITC assay. The ITC assay was performed using the auto-iTC200 instrument (MicroCal, GE). MDM2 protein or VEGF 3′UTR was loaded into a 96-well deep-well PP plate, and then the compound was titrated stepwise into the protein or RNA sample cell using a syringe, for a total of 16 injections. The equilibrium time between two adjacent injections was 210 s. The binding stoichiometry (n), binding constant (Kd) and thermodynamic parameters (ΔH and ΔS) were determined by fitting the titration curve to a one-site binding mode, using the Origin software provided by the manufacturer.

### Polysome preparation and analysis

Polysome profiling was carried out as described previously [12]. Cells were incubated with 100 μg/ml cycloheximide (CHX) for 15 minutes to arrest polyribosome migration. The cells were then lysed to isolate cytoplasmic RNA in a buffer containing 20 mM Tris–HCl at pH 8.0, 100 mM NaCl, 5 mM MgCl_2_, 0.5% Triton X-100, 500 U/ml RNAsin and a cocktail of protease inhibitors. The cell lysates were fractionated on a 15–45% (wt/vol) sucrose gradient and centrifuged in a SW41Ti rotor at 39,000 rpm for 2 h. Fractions from each gradient tube were collected by upward replacement, and absorption at optical density (OD)_254_ was monitored using a fractionator. The RNA in each fraction was extracted and subjected to quantitative RT-PCR.

### Ubiquitination assay

Cells were treated with MG132 (30 μM) for 4 h before collection. The cell pellet was lysed and incubated with Ni^2+^-NTA beads (Qiagen, Valencia, CA, USA) at room temperature for 4 h. The beads were then washed sequentially for 5 minutes in each of the following buffers: buffer A (6 M guanidinium-HCl, 0.1 M Na_2_HPO_4_/NaH_2_PO_4_, 0.01 M Tris–HCl (pH 8.0), 5 mM imidazole, 10 mM β-mercaptoethanol); buffer B (8 M urea, 0.1 M Na_2_HPO_4_/NaH_2_PO_4_, 0.01 M Tris–HCl (pH 8.0), 10 mM β-mercaptoethanol); buffer C (8 M urea, 0.1 M Na_2_HPO_4_/NaH_2_PO_4_, 0.01 M Tris–HCl (pH 6.3), 10 mM β-mercaptoethanol). This ubiquitinated product was finally eluted with buffer D (200 mM imidazole, 0.15 M Tris–HCl (pH 6.7), 30% glycerol, 0.72 M β-mercaptoethanol, 5% SDS) and analyzed by western blot using an anti-ubiquitin antibody (Cell Signaling).

### WST-1 cytotoxicity assay

The cytotoxic effect of gossypol was determined by WST-1 assay. Cells were cultured in 96-well plates along with different concentrations of gossypol for 24 h. WST-1 reagent (25 μg/well; Roche) was then added and incubation continued for an additional 4 h. Following this, the OD of the wells was read with a microplate reader (BioTek Instruments, Winooski, VT, USA). Appropriate controls lacking cells were included to determine background absorbance.

### Flow cytometry

Flow cytometry was conducted to analyze the ability of gossypol to induce apoptosis. Cells with or without gossypol treatment were stained with fluorescein isothiocyanate (FITC)-Annexin V and propidium iodide (BD Pharmingen, San Diego, CA, USA). The stained cells were then detected on a FACScan flow cytometer using the CellQuest software (BD Biosciences, Mountain View, CA, USA).

### Xenograft model

This study was approved by the Animal Care and Use Committee of Huazhong University of Science and Technology. Breast cancer xenograft models were established. After 7 days, tumors were detected and gossypol (dissolved in DMSO) was administrated intraperitoneally at a dose of 10 mg/kg/day for 4 weeks. Control group received control solution containing the same amount of DMSO. Tumor growth and angiogenesis, as determined by microvessel density (MVD), were evaluated for each of these xenograft tumors.

### Statistical analysis

At least three independent experiments were performed. All data were presented as mean ± SEM. Relative gene expression data were analyzed using the 2 − ΔΔCT method [24]. The statistical significance of differences between groups was determined by one-way analysis of variance (ANOVA) or by the nonparametric Kruskal-Wallis test with the use of SPSS 13.0 software. All statistical tests were two-sided. The significance level was set at *P* < 0.05.

## Results

### Gossypol binds to MDM2 RING protein to disrupt its interaction with VEGF 3′UTR, inhibiting the expression of both MDM2 and VEGF

**Fig. 1:**
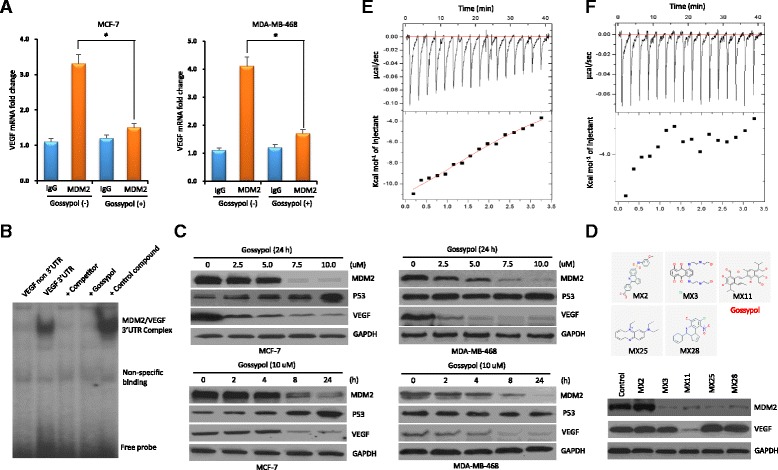
Gossypol disrupts the binding of mouse double minute 2 (*MDM2*) RING protein to vascular endothelial growth factor (*VEGF*) 3′UTR to inhibit the expression of MDM2 and VEGF in human breast cancer cell lines. **a** Protein-RNA binding assay to detect the effect of gossypol on the interaction between MDM2 protein and VEGF mRNA. MCF-7 and MDA-MB-468 cells were treated with or without gossypol before harvesting. Following co-immunoprecipitation with anti-MDM2, VEGF mRNA was detected by quantitative RT-PCR analysis. **b** UV crosslinking and RNA binding assay for testing the effect of gossypol on the interaction between MDM2 RING protein and VEGF 3′UTR (labeled with ^32^P). Controls include VEGF non-3′UTR probe, competitor (25-fold molar excess of unlabeled VEGF 3′UTR), and control small-molecule compound (no MDM2 inhibition). **c** Western blot assays showing the dose–response and time-course of MDM2 and VEGF inhibition by gossypol. MCF-7 and MDA-MB-468 cells were treated with different concentrations of gossypol for 24 h and 10 μM gossypol for different times, respectively. The expression of MDM2, p53 and VEGF were detected by western blot. Glyceraldehyde-3-phosphate dehydrogenase (*GAPDH*) served as an internal control for equal protein loading. **d** Comparison of the effects of gossypol (labeled MX11) and other compounds (labeled MX2, MX3, MX25 and MX28) on MDM2 and VEGF expression in MDA-MB-468 cells. **e**, **f** Determination of gossypol binding to MDM2 RING protein (**e**) or VEGF 3′UTR (**f**) as detected by isothermal titration calorimetry. The *upper box* is raw heating power over time and the *lower box* is a fit of integrated energy values, normalized for each injection

We first performed a protein-RNA binding assay to confirm whether gossypol could disrupt in vivo interaction between MDM2 protein and VEGF mRNA. To determine if this effect is dependent on p53, we compared the results in the paired MCF-7 (p53-wt) and MDA-MB-468 (p53-mutant) cells. Results from co-immunoprecipitation and quantitative RT-PCR analysis showed that the MDM2 protein was able to bind VEGF mRNA; however, gossypol disrupted their binding. As seen in Fig. 1a, in both MCF-7 and MDA-MB-468 cells, when precipitated with MDM2 antibody, the level of VEGF mRNA was significantly reduced after only 30 minutes of treatment with gossypol. More specifically, UV crosslinking and RNA binding assay showed that gossypol disrupted the binding of MDM2 RING protein to VEGF 3′UTR (Fig. 1b).

We then investigated the effect of gossypol on MDM2 and VEGF expression. We found that, regardless of the p53 status, gossypol significantly inhibited the cellular expression of both MDM2 and VEGF. Further, gossypol inhibited expression of both MDM2 and VEGF in a dose-dependent and time-dependent manner (Fig. 1c). As controls, other compounds (labeled MX2, MX3, MX25, and MX28) identified as the top hits that inhibit protein-RNA binding activity [22] did not simultaneously inhibit the expression of both MDM2 and VEGF (Fig. 1d).

In addition, the ITC assay was performed to determine whether gossypol binds to either MDM2 RING protein or to VEGF 3′UTR to disrupt their interaction. The results showed that gossypol bound to the MDM2 RING protein with a binding K_d_ value of 5.21 μM (Fig. 1e), but not to the VEGF 3′UTR (Fig. 1f). Therefore, gossypol binds to MDM2 RING protein to disrupt its interaction with VEGF 3′UTR and consequently inhibit the expression of both MDM2 and VEGF.

### Gossypol induces MDM2 self-ubiquitination and protein-degradation

**Fig. 2:**
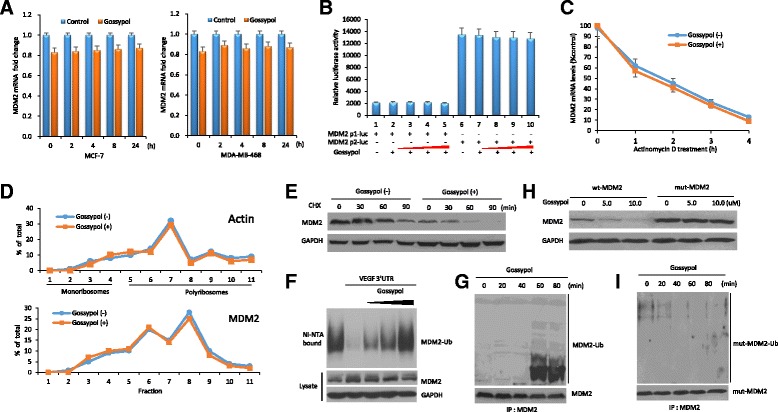
Gossypol induces mouse double minute 2 (*MDM2*) self-ubiquitination and protein-degradation. **a** MCF-7 and MDA-MB-468 cells were treated with 10 μM gossypol for different time periods. The mRNA levels of MDM2 in tumor cells were determined by quantitative RT-PCR. Data represent means ± SEM of three independent experiments normalized to glyceraldehyde-3-phosphate dehydrogenase (*GAPDH*). **b** MDA-MB-468 cells were transfected with MDM2 promoter luciferase plasmids (MDM2 p1-Luc or p2-Luc). Transfected cells were treated with increasing concentrations of gossypol (2.5, 5.0, 7.5, and 10.0 μM) for 4 h and then analyzed by luciferase activity assay. **c** MDA-MB-468 cells were treated with or without 10 μM gossypol for 4 h, followed by addition of 5 mg/ml actinomycin D. At different time points indicated after addition of the mRNA synthesis inhibitor actinomycin D, the cells were harvested and the amount of MDM2 mRNA in the cells was detected by quantitative RT-PCR. **d** MDA-MB-468 cells were treated with or without 10 μM gossypol for 8 h, and then their cytoplasmic lysates were fractionated on a sucrose gradient. RNA extracted from each of the fractions was subjected to quantitative RT-PCR for quantitative analysis of the distribution of MDM2 mRNA. Data represent percentage of the total amount of corresponding mRNA for each fraction. **e** MDA-MB-468 cells were treated with or without 10 μM gossypol for 4 h, followed by addition of a protein synthesis inhibitor cycloheximide (CHX, 50 μg/ml). At different time points after CHX, cell lysates were prepared and analyzed by western blot assay. **f** Ubiquitination assay in MDA-MB-468 cells for testing the effects of increasing concentrations of gossypol (2.5, 5.0, and 10.0 μM) on ubiquitination of MDM2 in the presence or absence of VEGF 3′UTR. **g** MDA-MB-468 cells were treated with a proteasome inhibitor MG132 (30 μM) for 4 h and subsequently with 10 μM gossypol for the indicated times. MDM2 ubiquitination was analyzed by immunoprecipitation-western blot assay. **h**, **i** Immunoprecipitation-western blot assay to detect the effect of gossypol on the ubiquitination of mut-MDM2 C464A without E3 ligase activity. *VEGF* vascular endothelial growth factor, *wt* wild-type

We investigated further how MDM2 is inhibited by gossypol. First, we performed quantitative RT-PCR for the expression of MDM2 mRNA in gossypol-treated cells. Gossypol did not inhibit MDM2 mRNA expression; instead, the MDM2 mRNA level actually increased in the p53-wt cell line (Fig. 2a). This was consistent with p53 activation as shown in Fig. 1c (the level of wt p53 was elevated in MCF-7 cells, while no significant effect on mutant p53 was observed in MDA-MB-468 cells). These results suggest that induction of MDM2 mRNA by gossypol could be attributed to p53 activation. A reporter assay in the p53-mutant MDA-MB-468 cell line further confirmed that gossypol did not directly regulate MDM2 transcription (Fig. 2b). Pulse-chase and quantitative RT-PCR indicated that the stability of MDM2 mRNA was not affected by gossypol (Fig. 2c). Results from polyribosome profiling showed that gossypol also did not regulate MDM2 translation (Fig. 2d). By CHX pulse-chase assay, we found that MDM2 inhibition by gossypol is through a protein-degradation mechanism. The half-life of MDM2 protein in control cells was more than 90 minutes, whereas gossypol treatment decreased the half-life of MDM2 to less than 30 minutes (Fig. 2e).

It is well-known that MDM2 is ubiquitinated through its own E3 ubiquitin ligase activity [25]. To further define the mechanism by which gossypol promotes MDM2 protein degradation, the possibility that gossypol could induce MDM2 self-ubiquitination was studied. As expected, gossypol indeed induced ubiquitination of endogenous MDM2, which was associated with VEGF 3′UTR (Fig. 2f, g). This ubiquitination required the intrinsic self-ubiquitination E3 ligase activity of MDM2 itself. While gossypol induced degradation and ubiquitination of wt MDM2, it was unable to induce degradation and ubiquitination of the C464A mutant MDM2 that lacks E3 ubiquitin ligase activity [26] (Fig. 2h, i). Therefore, gossypol inhibits MDM2 expression through induction of MDM2 self-ubiquitination and protein-degradation.

### Gossypol decreases VEGF mRNA stability and thereby its protein translation

**Fig. 3:**
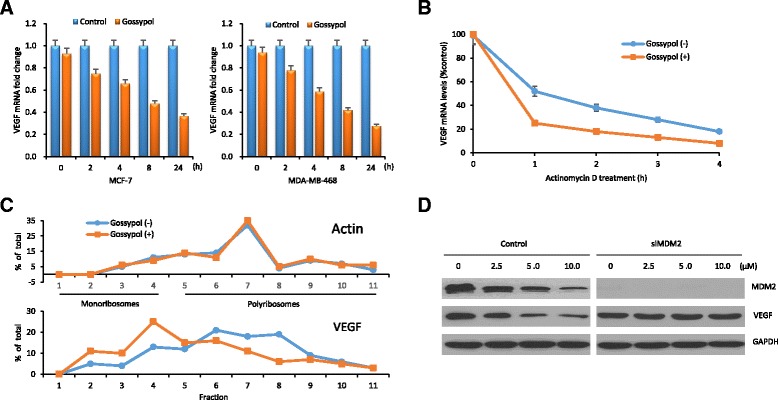
Gossypol decreases vascular endothelial growth factor (*VEGF*) mRNA stability and thereby its protein translation. **a** Quantitative RT-PCR for VEGF mRNA expression in MCF-7 and MDA-MB-468 cells treated with 10 μM gossypol for different time periods. **b** Pulse-chase and quantitative RT-PCR for the stability of VEGF mRNA in MDA-MB-468 cells treated with gossypol. **c** MDA-MB-468 cells were treated with or without 10 μM gossypol for 8 h, and then their cytoplasmic lysates were fractionated on a sucrose gradient. RNA extracted from each of the fractions was subjected to quantitative RT-PCR for quantitative analysis of the distribution of VEGF mRNA. Data represent percentage of the total amount of corresponding mRNA for each fraction. **d** Western blot for expression of MDM2 and VEGF in MDA-MB-468 cells with MDM2 knockdown treated with indicated doses of gossypol for 24 h

When we evaluated the mechanism by which gossypol inhibits VEGF, we found that gossypol decreased VEGF mRNA stability and thereby its protein translation. Gossypol inhibited the VEGF mRNA level (Fig. 3a), which was due to decreased mRNA stability (Fig. 3b). The half-life of VEGF mRNA in MDA-MB-468 cells with gossypol treatment (T_1/2_ = 38 ± 4 minutes) was significantly shorter than that in untreated cells (T_1/2_ = 65 ± 7 minutes).

Because of decreased mRNA stability, we took steps to investigate whether there also was a decrease in VEGF protein synthesis. We performed linear sucrose-gradient fractionation to assess the association between polyribosomes and VEGF mRNA in MDA-MB-468 cells subjected to gossypol treatment (Fig. 3c). We found that VEGF mRNA shifted away from fractions enriched with translating polyribosomes to fractions containing translation-dormant complexes, which is indicative of decreased translation. In contrast, gossypol had no effect on the polyribosome profile of a control glyceraldehyde-3-phosphate dehydrogenase (GAPDH) mRNA.

We also investigated whether inhibition of VEGF is associated with MDM2. Gossypol inhibited VEGF in control MDA-MB-468 cells but not in MDA-MB-468 cells with MDM2 knockdown (Fig. 3d), suggesting that gossypol-mediated inhibition of VEGF is MDM2-dependent.

### Gossypol induces cancer cell apoptosis, dependent on MDM2 inhibition

**Fig. 4:**
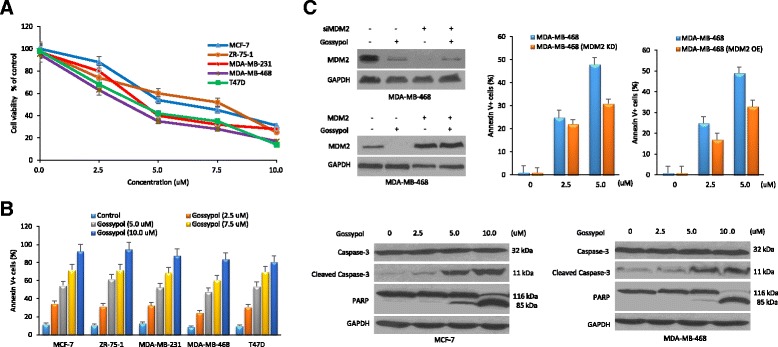
Cytotoxic and apoptotic effects of gossypol on human breast cancer cells. Cells were treated with different concentrations of gossypol for 24 h for cell viability (**a**) and apoptosis assay (**b**). All assays were performed in triplicate. **c** MDA-MB-468 cells were transfected with mouse double minute 2 (*MDM2*) siRNA or overexpression plasmid, and then the tumor cells were incubated with different concentrations of gossypol for 24 h followed by cell apoptosis assay. Cells with MDM2 knockdown or overexpression were less responsive to gossypol-induced apoptotic effect than control cells. *GAPDH* glyceraldehyde-3-phosphate dehydrogenase, *PARP* poly(ADP-ribose) polymerase

Next, we investigated the effects of gossypol on cancer cell viability and apoptosis. We studied five human breast cancer cell lines, with different p53 status (MCF-7 and ZR-75-1/p53-wt; MDA-MB-231, MDA-MB-468 and T47D/p53-mutant). WST-1 assay showed that gossypol exhibited significant cytotoxic activity against all these cell lines (Fig. 4a).

To clarify whether the observed cell death was associated with induction of apoptosis, gossypol-treated cancer cells were stained with Annexin V and quantitated by flow cytometry. We found that gossypol-induced cell death was indeed due to apoptosis. Gossypol induced apoptosis in both wt and mutant p53-expressing cancer cells. As the concentration of gossypol increased, the percentage of apoptotic cells gradually increased. Correspondingly, cleavage of caspase-3 and PARP was detected by western blotting after gossypol treatment (Fig. 4b).

We ask how critical oncoprotein MDM2 is to the gossypol-induced cell apoptosis and death. Results as shown in Fig. 4c indicated the importance of MDM2 inhibition. MDM2 siRNA or overexpression plasmid was introduced into MDA-MB-468 cells. Cells with MDM2 knockdown or overexpression were less responsive to gossypol-induced apoptotic effect than control cells.

### Gossypol suppresses tumor growth and VEGF-mediated angiogenesis in breast cancer xenograft models

**Fig. 5:**
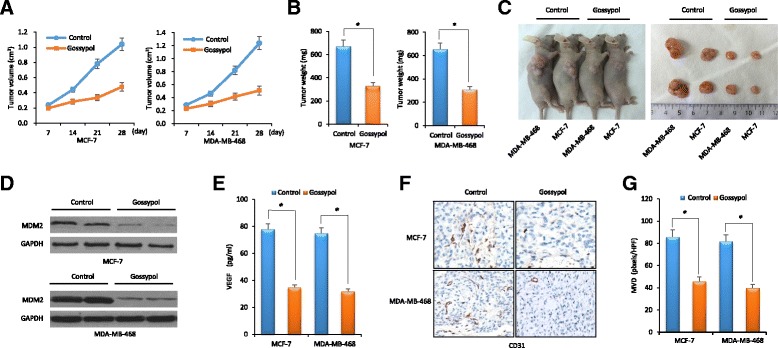
Gossypol suppresses tumor growth and vascular endothelial growth factor (VEGF)-mediated angiogenesis in breast cancer xenograft models. MCF-7 or MDA-MB-468 cells were subcutaneously implanted into nude mice and subsequently treated with gossypol. All mice underwent monitoring of tumor growth. On day 42 post inoculation, serum samples and xenograft tumor tissues were obtained for MDM2 expression, VEGF production and neovascularization analysis. **a** Xenograft tumor size. **b** Xenograft tumor weight on day 42. **c** Representative pictures from each group. **d** Expression of Mouse double minute 2 (*MDM2*) in tumor tissues determined by western blot. **e** Concentrations of VEGF in the serum samples of nude mice determined by ELISA. **f** Representative histology comparisons of tumor vessel content in xenografts from different treatment group, as stained by CD31 immunohistochemistry (×200). **g** Positive immunostaining of CD31 was digitally quantified and measured as microvessel density (*MVD*). Data represent mean ± SEM; *n* = 10 mice/group. *P < 0.05. *GAPDH* glyceraldehyde-3-phosphate dehydrogenase

Given the observed inhibitory effect of gossypol on MDM2 and VEGF expression and cancer cell apoptosis, we investigated whether gossypol could suppress tumor growth and VEGF-mediated angiogenesis in a nude mouse xenograft model. The breast cancer xenograft model was established and treated by intraperitoneal injections of gossypol. Tumor growth was continuously monitored after treatment. Our results showed that in both the MCF-7 and MDA-MB-468 xenograft models, gossypol treatment significantly suppressed the growth of the xenograft tumor. Compared to control tumors, gossypol suppressed the xenograft tumor growth by 50.6% in the MCF-7 xenograft model and by 53.0% in the MDA-MB-468 xenograft model, respectively (Fig. 5a–c).

Upon processing tumor tissues, we found that gossypol inhibited in vivo expression of MDM2 (Fig. 5d). In addition, serum levels of VEGF in nude mice were measured by ELISA (Fig. 5e), providing further evidence for the simultaneous inhibition of VEGF production by gossypol. These results were consistent with those observed in vitro in the breast cancer cell lines treated with gossypol. Finally, the effect of gossypol on tumor angiogenesis was evaluated by immunostaining for the endothelial cell marker CD31. Positive staining was digitally quantified and measured as MVD. Anti-angiogenesis activity occurred following administration of gossypol. Compared to control, there was a significant decrease in CD31 staining (Fig. 5f). Accordingly, there was significant suppression of MVD by 46.5% in the MCF-7 xenograft model and by 51.2% in the MDA-MB-468 xenograft model, respectively (Fig. 5g).

## Discussion

There is evidence of the anti-cancer effects of gossypol [17], and several studies have investigated the possible molecular targets and mechanisms of action. For example, gossypol has been identified as a Bcl-2 homology 3 (BH3)-mimetic, is able to bind to the BH3 domain of Bcl-2 family members, and induces apoptosis [18]. In addition, gossypol modulates the p53 [19], c-Myc [20] and NF-κB [21] signaling pathways. In the present study, we reported that gossypol is a potent inhibitor of MDM2-VEGF. Through disrupting the molecular interaction between MDM2 protein and VEGF mRNA, gossypol induces MDM2 self-ubiquitination and decreases VEGF translation, which results in both apoptosis and anti-angiogenesis effects.

Our study revealed a novel mechanism by which gossypol functions as an anticancer agent. MDM2 and VEGF are important molecules involved in tumor progression. MDM2 acts as an oncoprotein, while VEGF plays crucial roles in tumor angiogenesis. For years, our research has focused on the p53-independent activity and mechanism of MDM2 in the regulation of VEGF [12]. We also try to screen for potential inhibitors that simultaneously target MDM2 and VEGF. Although cytotoxic drugs kill tumor cells, the plentiful blood supply to the tumor can still allow residual tumor cells to continue growing. Therefore, we believe that MDM2-VEGF dual-targeting represents a novel cancer therapy strategy, not only against cancer cells, but also against the tumor microenvironment, especially tumor angiogenesis. This will offer greater control on tumor growth.

For the first time, we described the inhibition of gossypol on MDM2. Compared with other classical MDM2 inhibitors like nutlin-3a, gossypol is distinct in that it inhibits MDM2 and induces apoptosis in cancer cells with a different p53 background. As mentioned, MDM2-p53 interaction inhibitors have several limitations. First, these inhibitors exhibit effects only in cancer cells bearing wild-type p53. Second, these inhibitors do not inhibit MDM2, and expression of MDM2 can even be enhanced through the p53-MDM2 feedback regulatory loop. Third, these inhibitors would not be able to inhibit the p53-independent activities of MDM2. Therefore, gossypol should be particularly useful, especially for high-risk, refractory cancer patients. Studies have shown that most cancer patients, including breast cancer patients, do not express wild-type p53 and mutations in p53 predict worse prognosis and poor treatment outcome [27, 28].

Herein, a mechanistic study indicated that gossypol induces MDM2 self-ubiquitination and protein degradation. MDM2 has been well-characterized as a member of the RING-finger-type family of E3 ubiquitin ligases. MDM2 regulates ubiquitination of not only p53 but also MDM2 itself [25]. The E3 ligase activity responsible for MDM2 self-ubiquitination is regulated by many cellular signaling pathways and molecular events. Previous studies showed that nucleic acids, such as polyA, polyG and certain cellular small RNA, are able to bind to the RING domain of MDM2 and suppress its self-ubiquitination activity [29, 30]. In the current study, the dissociation between MDM2 protein and VEGF mRNA following treatment with gossypol led not only to decreased translation of VEGF mRNA but also to simultaneous self-ubiquitination of MDM2 protein. These results provide a preliminary indication that binding of VEGF mRNA to MDM2 protein may also suppress its self-ubiquitination activity, which results in increased MDM2 protein stability and expression. We have shown that, in response to hypoxia, MDM2 protein is translocated from the nucleus to the cytoplasm. In the cytoplasm, MDM2 binds to VEGF mRNA and increases VEGF mRNA stability and translation [12]. On the other hand, further detailed studies are required to give direct evidence that MDM2 and VEGF are mutually regulated, and the E3 ubiquitin-ligase activity of the MDM2 RING protein for self-ubiquitination is negatively regulated by the binding of the VEGF mRNA.

VEGF is the most prominent angiogenic factor that plays crucial roles in tumor angiogenesis. Gossypol has previously been reported to modulate the VEGF signaling pathway [31]. Gossypol inhibits VEGF expression in human prostate cancer cells. Further, gossypol blocks multiple steps in VEGF-activated biological events in endothelial cells. For example, gossypol inhibits endothelial cell viability, chemotactic motility, and microvessel sprouting. Gossypol exerts its function through blockade of the VEGF/VEGFR2 signal cascade in vascular endothelial cells. In agreement, in this study, we also observed that there was significant inhibition of VEGF in human breast cancer following treatment with gossypol. And besides that, importantly, we illuminated the mechanism by which gossypol inhibits production of VEGF by cancer cells. Gossypol decreases VEGF mRNA stability and thereby its protein translation. Our results help to better understand the functional property of gossypol in the VEGF signaling pathway.

## Conclusions

In summary, gossypol is a dual inhibitor of MDM2 and VEGF that disrupts the molecular interaction between MDM2 protein and VEGF mRNA, induces MDM2 self-ubiquitination and degradation, decreases VEGF mRNA stability and protein translation simultaneously, and therefore exerts anti-cancer effects through apoptotic and anti-angiogenesis pathways in human breast cancer in vitro and in vivo, regardless of the p53 status of the cancer cells. We believe development of these MDM2-VEGF inhibitors as potential anticancer drugs for clinical use is worthwhile and represents a novel strategy for improving cancer outcome.

## Abbreviations

CHX: Cycloheximide
DMSO: Dimethyl sulfoxide
ELISA: Enzyme-linked immunosorbent assay
FP: Fluorescence polarization
GAPDH: Glyceraldehyde-3-phosphate dehydrogenase
HTS: High-throughput screening
ITC: Isothermal titration calorimetry
MDM2: Mouse double minute 2
MVD: Microvessel density
PARP: Poly(ADP-ribose)
UTR: Untranslated region
VEGF: Vascular endothelial growth factor
wt: wild-type
